# Identification and characterization of long intergenic noncoding RNAs in bovine mammary glands

**DOI:** 10.1186/s12864-017-3858-4

**Published:** 2017-06-19

**Authors:** Chao Tong, Qiaoling Chen, Lili Zhao, Junfei Ma, Eveline M. Ibeagha-Awemu, Xin Zhao

**Affiliations:** 1 College of Animal Science and Technology, Northwest A&F University, Yangling, Shaanxi People’s Republic of China; 2 Agriculture and Agri-Food Canada, Research and Development Centre, Sherbrooke, QC J1M 0C8 Canada; 3 Department of Animal Science, McGill University, 21111 Lakeshore Road, Ste-Anne-De-Bellevue, QC H9X 3V9 Canada

**Keywords:** Bovine mammary gland, LincRNAs, Quantitative trait loci

## Abstract

**Background:**

Mammary glands of dairy cattle produce milk for the newborn offspring and for human consumption. Long intergenic noncoding RNAs (lincRNAs) play various functions in eukaryotic cells. However, types and roles of lincRNAs in bovine mammary glands are still poorly understood.

**Results:**

Using computational methods, 886 unknown intergenic transcripts (UITs) were identified from five RNA-seq datasets from bovine mammary glands. Their non-coding potentials were predicted by using the combination of four software programs (CPAT, CNCI, CPC and hmmscan), with 184 lincRNAs identified. By comparison to the NONCODE2016 database and a domestic-animal long noncoding RNA database (ALDB), 112 novel lincRNAs were revealed in bovine mammary glands. Many lincRNAs were found to be located in quantitative trait loci (QTL). In particular, 36 lincRNAs were found in 172 milk related QTLs, whereas one lincRNA was within clinical mastitis QTL region. In addition, targeted genes for 10 lincRNAs with the highest fragments per kilobase of transcript per million fragments mapped (FPKM) were predicted by LncTar for forecasting potential biological functions of these lincRNAs. Further analyses indicate involvement of lincRNAs in several biological functions and different pathways.

**Conclusion:**

Our study has provided a panoramic view of lincRNAs in bovine mammary glands and suggested their involvement in many biological functions including susceptibility to clinical mastitis as well as milk quality and production. This integrative annotation of mammary gland lincRNAs broadens and deepens our understanding of bovine mammary gland biology.

**Electronic supplementary material:**

The online version of this article (doi:10.1186/s12864-017-3858-4) contains supplementary material, which is available to authorized users.

## Background

With the rapid development of sequencing technologies, especially the next-generation sequencing, a large number of transcripts have been accumulated through the RNA sequencing technology (RNA-seq). Some of these transcripts are noncoding RNAs (ncRNAs) and they may have diverse functions in biological processes. MicroRNAs, tRNA halves (tiRNAs) and Piwi-interacting RNA (piRNAs) are short ncRNAs with lengths of less than 200 bp, while ncRNAs longer than 200 bp are called long noncoding RNAs (lncRNAs). Based on locations of lncRNAs, they can be classified as antisense lncRNAs, intronic lncRNAs, bidirectional lncRNAs, intergenic lncRNAs (lincRNAs) and sense-overlapping lncRNAs.

In recent years, a growing number of lncRNAs have been discovered in eukaryotic organisms. In particular, lincRNAs and lincRNA candidates have been catalogued for human, mouse, zebrafish, frog, fly, nematode, arabidopsis, maize, yeast, pig, chicken and plasmodium [1, 2]. These lincRNAs are an important component of the regulatory architecture and they are involved in chromatin modification, epigenetic regulation, genomic imprinting, transcriptional control as well as pre- and posttranslational mRNA processing [1, 3].

Mammary glands have cycles of development and regression including pregnancy, lactation and involution throughout an adult female life. The role of lncRNAs in development and differentiation of mammary glands have been studied in a few studies. The pregnancy induced noncoding RNA (*PINC*) is one of first reported lncRNAs having the function in regulating development of mouse mammary epithelial cells. Mouse *PINC1.0* and mouse *PINC1.6* are two splice forms in mouse mammary epithelial HC11 cells. Knockdown of *PINC1.0* led to apoptosis, while knockdown of *PINC1.6* induced cell proliferation in HC11 cells, respectively [4]. In addition, *Neat1* (nuclear-enriched abundant transcript 1) is an abundant lncRNA and conserved in the mammalian lineage [5]. It also plays a key role in mammary gland development. Loss of *Neat1* reduced numbers of luminal alveolar epithelial cells and influenced normal mammary gland development in mice [6]. Another lncRNA *Zfas1* was highly expressed in primary mammary epithelial cells from pregnant mice and knockdown of *Zfas1* increased the proliferation rate of cells and induced beta-casein mRNA expression [7].

A few studies have reported existence of bovine lncRNAs, mainly in non-mammary gland tissues. Qu and Adelson identified 12,614 intergenic ncRNAs and 9337 intronic ncRNAs from public bovine Expressed Sequence Tags (ESTs) data [8]. Huang et al. predicted 449 putative lncRNAs which were located in 405 intergenic regions from bovine ESTs [9]. Weikard et al. detected lincRNAs in bovine skin samples (pigmented and non-pigmented) and identified 4848 potential lncRNAs with most of them being classified as lincRNAs [10]. Billerey et al. explored the lincRNA in Limousin bull muscle samples and found 584 different lincRNAs [11]. Finally, Koufariotis et al. catalogued a comprehensive list of putative bovine lncRNA located within intergenic and pseudogene regions which were expressed in 18 tissues including mammary glands [12].

To identify noncoding RNAs and their corresponding genes and to simplify the analysis to avoid the complications arising from overlap with other types of genes, recent focuses have been on lincRNA, which do not overlap exons of either protein-coding or other non-lincRNA types of genes [1]. Up to now, very few studies have specifically profiled lincRNAs in bovine mammary glands. Thus, the focus of our current study was to profile lincRNA transcripts of bovine mammary glands. We assembled transcripts from bovine mammary glands by an integrative approach. Four programs including the Coding-Non-Coding Index (CNCI) [13], the Coding Potential Calculator (CPC) [14], the Coding Potential Assessment Tool (CPAT) [15] and the hmmscan [16] were used to identify lincRNA candidates in mammary gland RNA-seq datasets. Using stringent criteria, 184 lincRNAs were found and expression of six randomly selected lincRNAs was confirmed by real-time PCR. One hundred and twelve novel lincRNAs were identified, contributing to the lincRNA repertoire. The potential relationships between lincRNAs and QTLs and between lincRNAs and signaling pathways in bovine mammary glands improve our understanding of lincRNAs in milk production and milk quantity.

## Methods

### Databases

Five sets of RNA-seq data were downloaded from the NCBI Sequence Read Archive (SRA) database (Additional file 1). These datasets include two single-read samples and three paired-end samples that were 36 to 100 bp long sequenced on Illumina platforms (~100 million reads for the total). The *Bos taurus* UMD3.1 reference genome FASTA file and the Gene Transfer Format (GTF) file were downloaded from the ensembl website (http://asia.ensembl.org). The UniRef90 (UniProt Reference Clusters) database was downloaded from the UniProt website (http://www.ebi.ac.uk/uniprot/database/download.html).

### Alignment of RNA-seq reads and assembly of transcripts

The quality control of downloaded RNA sequences was performed by the FastQC software (http://www.bioinformatics.babraham.ac.uk/projects/fastqc, version 0.11.2). Adaptors were filtered using the Trimmomatic program (http://www.usadellab.org/cms/?page=trimmomatic, version 0.33). RNA-seq reads from bovine mammary glands were aligned to the *Bos Taurus* UMD3.1 reference genome with the TopHat2 (version 2.0.12) [17]. Mapped reads were assembled with the Cufflinks (version 2.2.1) [18]. All assembled transcripts were then merged using the cuffcompare (version 2.2.1) [19].

### Identification of putative LincRNAs

There is no generally accepted or standard methodology that allows for easy discovery of lincRNAs. Thus, in order to identify true intergenic lincRNAs and avoid false-positive ones, stringent conditions were applied in this study to filter the mapped reads with the following criteria: (1) Only unknown intergenic transcripts (UITs) were used to identify putative lincRNAs; (2) If UITs had only one exon or the length of UITs was less than 200 bp, they were discarded [20]; (3) The UITs with low expression levels (fragments per kilobase of transcript per million fragments mapped, FPKM <1) and the minimal read coverage threshold of these transcript below three were discarded; (4) The UITs closest to the coding gene less than 1 kb were discarded; (5) To predict coding potentials of the remaining UITs which are not annotated in the bovine genome, four programs including the CNCI, the CPC, the CPAT and the hmmscan were used concurrently. The CPC (version 0.9-r2), a SVM based algorithm (http://cpc.cbi.pku.edu.cn/), uses UniRef90 to identify protein-coding UITs or noncoding UITs. We selected the coding potential score > 0 as the coding UITs and the coding potential score < 0 as the noncoding UITs. The CPAT (version 1.2.2) uses a logistic regression model to assess the coding or noncoding transcripts in our UITs. We downloaded 10,000 bovine known protein sequences from the ensembl website and 10,000 bovine noncoding RNAs from the NONCODE database (version 4) (http://www.noncode.org/index.php). These sequences were used for training by CPAT. The software calculated the hexamer tables and a bovine specific logistic regression model was built. Because the coding probability score from the CPAT was different in different species, the cut-off value of 0.348 was chosen for reliability and sensitivity according a previous similar study of cows [11]. The UITs with scores <0.348 were retained as putative noncoding RNAs . The CNCI (version 2) program profiles the adjoining nucleotide triplets (ANT) to differentiate coding and noncoding sequences. The CNCI software was downloaded from the web (https://github.com/www-bioinfo-org/CNCI) and used with the default setting. Finally, all 886 UITs were translated into six possible open reading (ORF) frames. Six possible ORF frames contain three frames for the sense strand and three frames for antisense strand. The six ORF frames were compared against the Gene3D, Pfam, TIGRFAM and Superfamily databases using the hmmscan algorithm. If one or more motifs were found in any of six possible ORF frames, the UIT was considered as a coding UIT and was discarded. Only noncoding UITs identified by all four software programs were considered as our putative lincRNAs.

### Localization of lincRNAs in quantitative trait loci

The positions of the 184 putative lincRNAs were compared with positions of know quantitative trait loci (QTL) on the *Bos taurus* UMD3.1 reference genome according to the AnimalQTLdb. The AnimalQTLdb is a public QTL database on animal species including cattle, chicken, horse, pig, rainbow trout and sheep (http://www.animalgenome.org/QTLdb/) [21].

### Prediction of lincRNA–RNA interactions and pathway analyses

In order to predict the targets of lincRNAs in mammary glands and thus understand potential functions of lincRNAs, the LncTar tool with the first type of file format for predicting the lincRNA–RNA interactions was used. The first type of file format contains two files. One file was lincRNA sequence file and the other file was mRNA sequence file. During the analysis, only lincRNAs with the lowest normalized free energy < −0.14 were selected as possible lincRNA target genes. Predicted gene targets were used for further analyses of gene ontology (GO) functional annotations and KEGG pathway analysis using the R package clusterProfiler [22].

### Validation by RT-PCR

The PCR primers for six randomly selected lincRNAs were designed by the Primer Premier 5 (PREMIER Biosoft international, Palo Alto, CA, USA). Primer sequences are in Additional file 2. Expected lengths of PCR products were from 206 to 336 bp. Total RNA was extracted from bovine mammary epithelial cells (MAC-T) by Trizol according to the manufacturer’s instructions (Invitrogen, Carlsbad, CA). The first strand of cDNA was synthesized using the PrimeScript™ RT reagent kit (Takara, Dalian, China) according to the manufacturer’s instructions. PCR was performed using the 2 × EasyTaq PCR SuperMix (TransGen Biotech, Beijing, China). The following PCR cycling condition was used: 94 °C 5 min, followed by 35 cycles of 94 °C for 30s, annealing for 30s (annealing temperatures for the lincRNAs are in Additional file 2), 72 °C for 30s and a final extension step was 72 °C for 10 min. Five ul of each PCR product was analyzed by 1% agarose gel.

## Result

### Alignment of RNA-seq reads and transcriptome assembly

To comprehensively and accurately identify lincRNAs in bovine mammary glands, five un-stranded RNA-seq datasets from bovine mammary glands were used in this study. A total of 104,893,960 reads were obtained after trimming. The reads were aligned to the *Bos taurus* UMD3.1 reference genome using the TopHat2. Transcripts were reconstructed using the Cufflinks. To get a unique set of isoforms for each transcript locus, all reconstructed transcripts were assembled using the Cuffcompare against the *Bos taurus* UMD3.1 reference genome. Consequently, 129,889 transcripts were assembled.

**Fig. 1:**
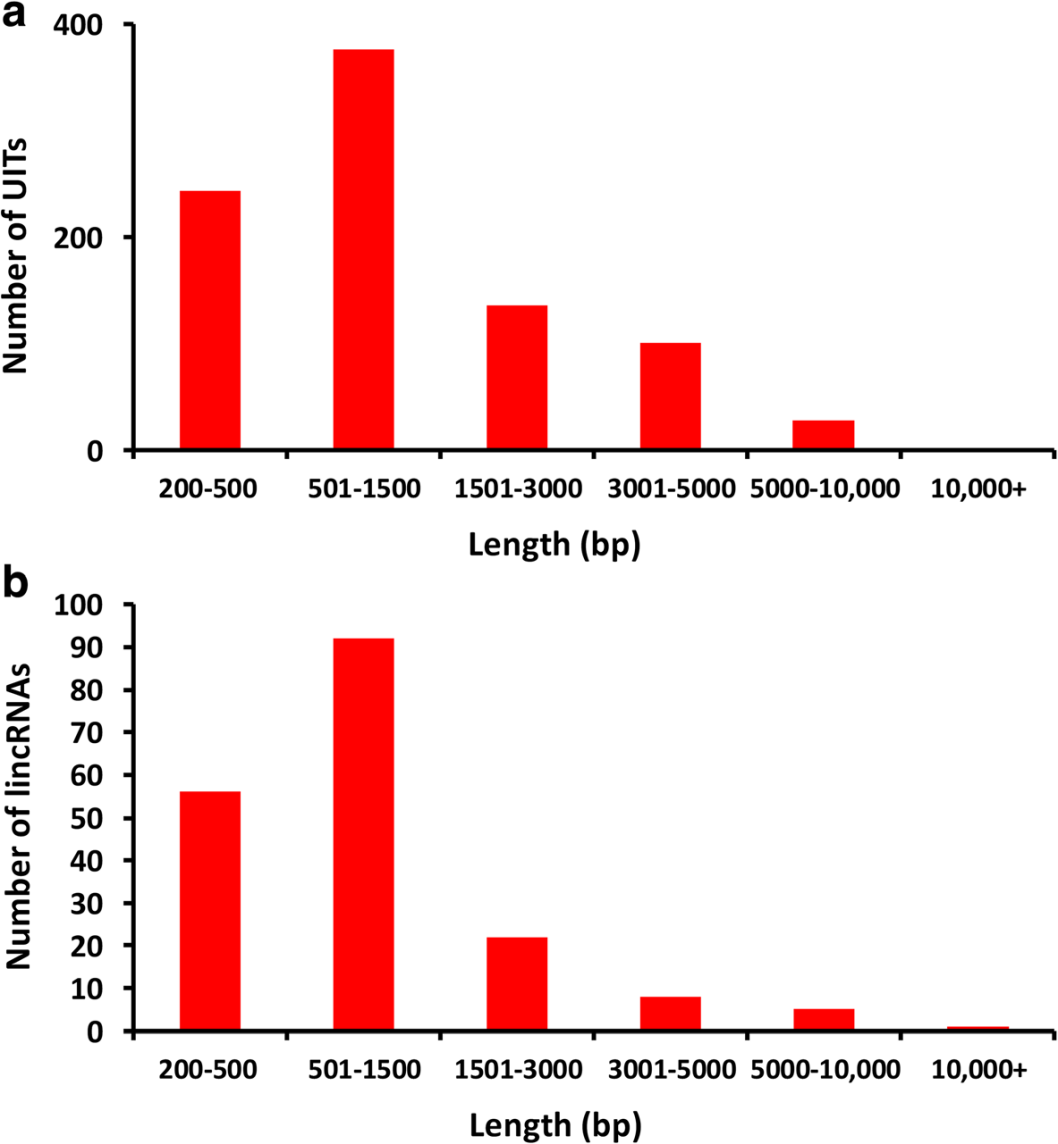
Length distribution and number of unknown intergenic transcripts (UITs) or lincRNAs. **a** Length distribution and number of 886 UITs, **b** Length distribution and number of 184 lincRNAs

Since reads were not assembled from strand-specific RNA-seq libraries, only UITs were used to identify putative lincRNAs in the bovine mammary glands. Very stringent conditions were used to filter assembled transcripts, as described in the materials and methods. After the filtering, only 886 UITs remained with sizes ranging from 204 to 13,156 bp. Among them, approximately 42% had a length ranging from 501 to 1500 bp (Fig. 1a).

**Fig. 2:**
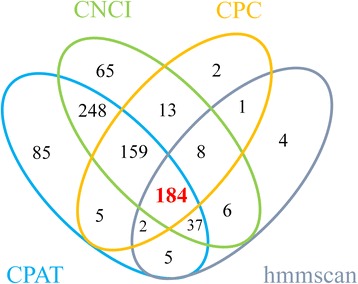
The number of lincRNA identified by each or combination of four programs

To discriminate noncoding UITs from coding ones, four software programs were applied to predict the protein-coding potential of 886 UITs. The CPC scores resulted in 374 putative noncoding UITs, while calculation by CPAT yielded 725 putative noncoding UITs. The CNCI analysis led to 720 putative noncoding UITs. Finally, 886 UITs were translated into six possible ORFs by the hmmscan algorithm against the Gene3D, Pfam, TIGRFAM and Superfamily databases. Six ORFs led to 247 putative noncoding UITs existing in all six ORFs. Based on the results from all four programs, 184 putative lincRNAs were identified (Fig. 2).

### Sizes and locations of identified lincRNAs

**Fig. 3:**
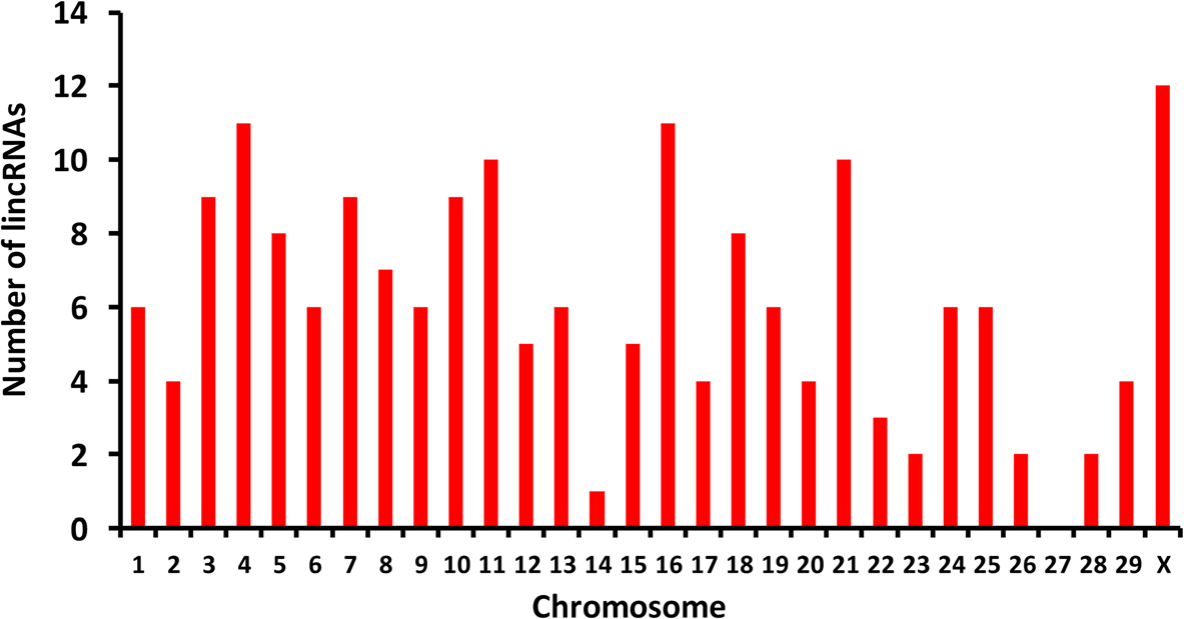
The number of 184 lincRNAs detected on each chromosome

The size of 184 putative lincRNAs ranged from 205 to 13,156 bp (Fig. 1b). The length variation of lincRNAs was similar to that of the UITs. Only one lincRNA (TCONS_00130959) was more than 10,000 bp long. The distribution of 184 lincRNAs on each chromosome was plotted in Fig. 3. LincRNAs were present, but not equally, on every chromosome except chromosome 27. The highest numbers of lincRNAs were found on chromosome X (12 lincRNAs), chromosome 4 (11 lincRNAs) and chromosome 16 (11 lincRNAs). Interestingly, two lincRNAs were located in *Bos taurus* unplaced genomic scaffolds. The BLASTN (V2.6.0+) was used to compare our predicted lincRNAs with NONCODE 2016 and ALDB databases [23, 24]. The standard for sequence similarity was adopted from a previous similar study [10] and was defined with a mapping identity of ≥70% and a total sequence identity of ≥75% in a covered region ≥100 nt. After the comparison of the two databases, 112 novel lincRNAs were detected (Additional file 3).

### Localizing lincRNAs in QTL-associated regions

**Fig. 4:**
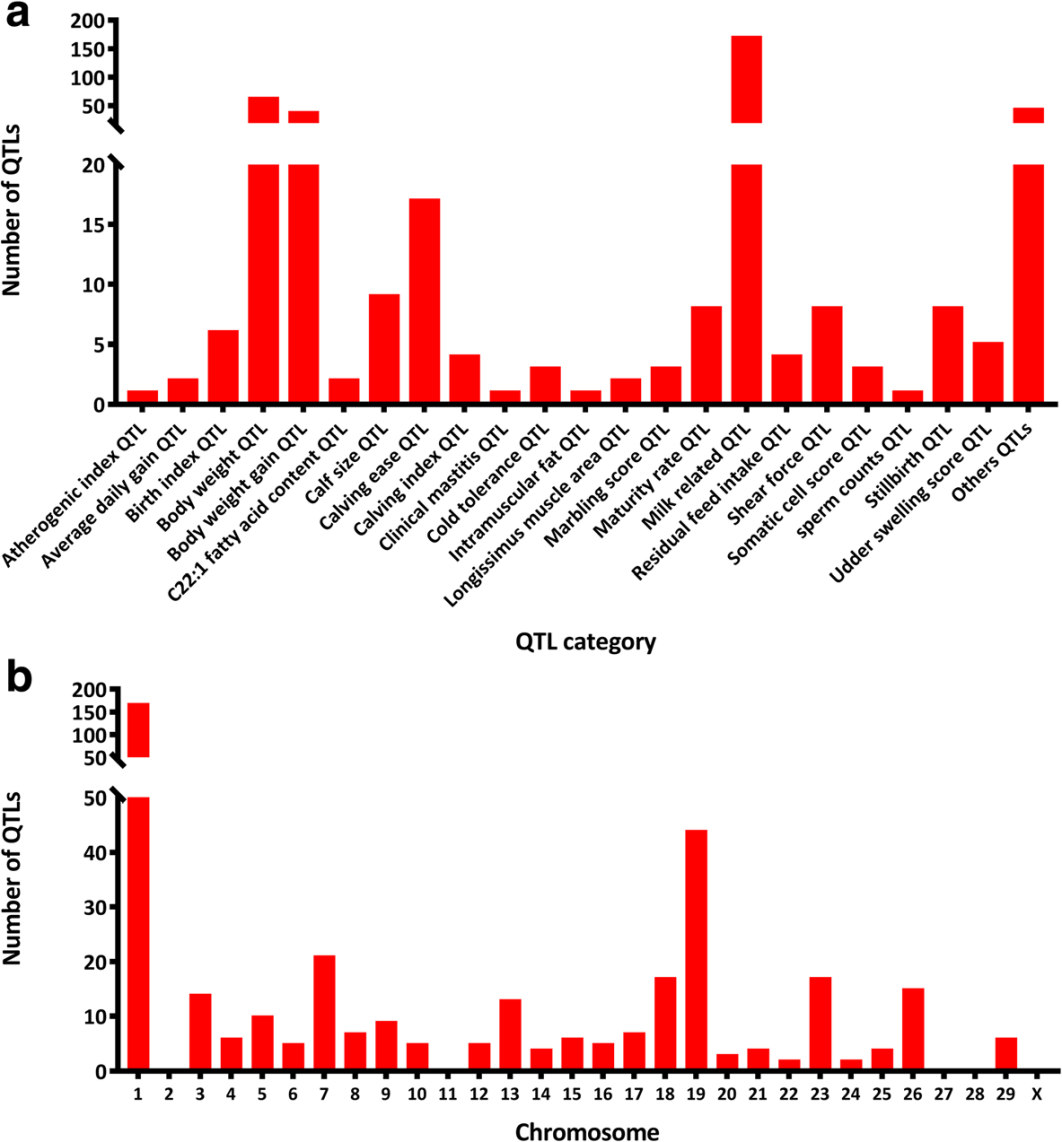
The category for all QTLs or QTLs detected on each chromosome. **a** The category for all QTLs, **b** QTLs detected on each chromosome. Milk related QTLs included Milk butyric acid percentage QTL, Milk capric acid percentage, Milk caprylic acid percentage, Milk conjugated linoleic acid percentage, Milk decenoic acid percentage QTL, Milk iron content QTL, Milk kappa-casein content QTL, Milk lauric acid percentage QTL, Milk lauroleic acid percentage QTL, Milk myristic acid percentage, Milk oleic acid percentage QTL, Milk palmitic acid percentage QTL, Milk phosphorus content QTL, Milk protein content QTL and Milk yield QTL

The AnimalQTLdb contains 17,908 cattle QTLs and 514 different cattle traits. By comparing locations of our 184 lincRNAs with QTL positions, 113 different lincRNAs were located in 399 cattle QTLs (Fig. 4a, Additional file 4). For example, one lincRNA was located in clinical mastitis QTLs and 36 lincRNAs in 169 unique milk related QTLs, suggesting that lincRNAs may be involved in mastitis susceptibility and regulation of milk yield and content (Additional file 4). The highest numbers of QTLs containing lincRNAs were found on chromosome 1 (Fig. 4b).

### Prediction of lincRNA functions

**Fig. 5:**
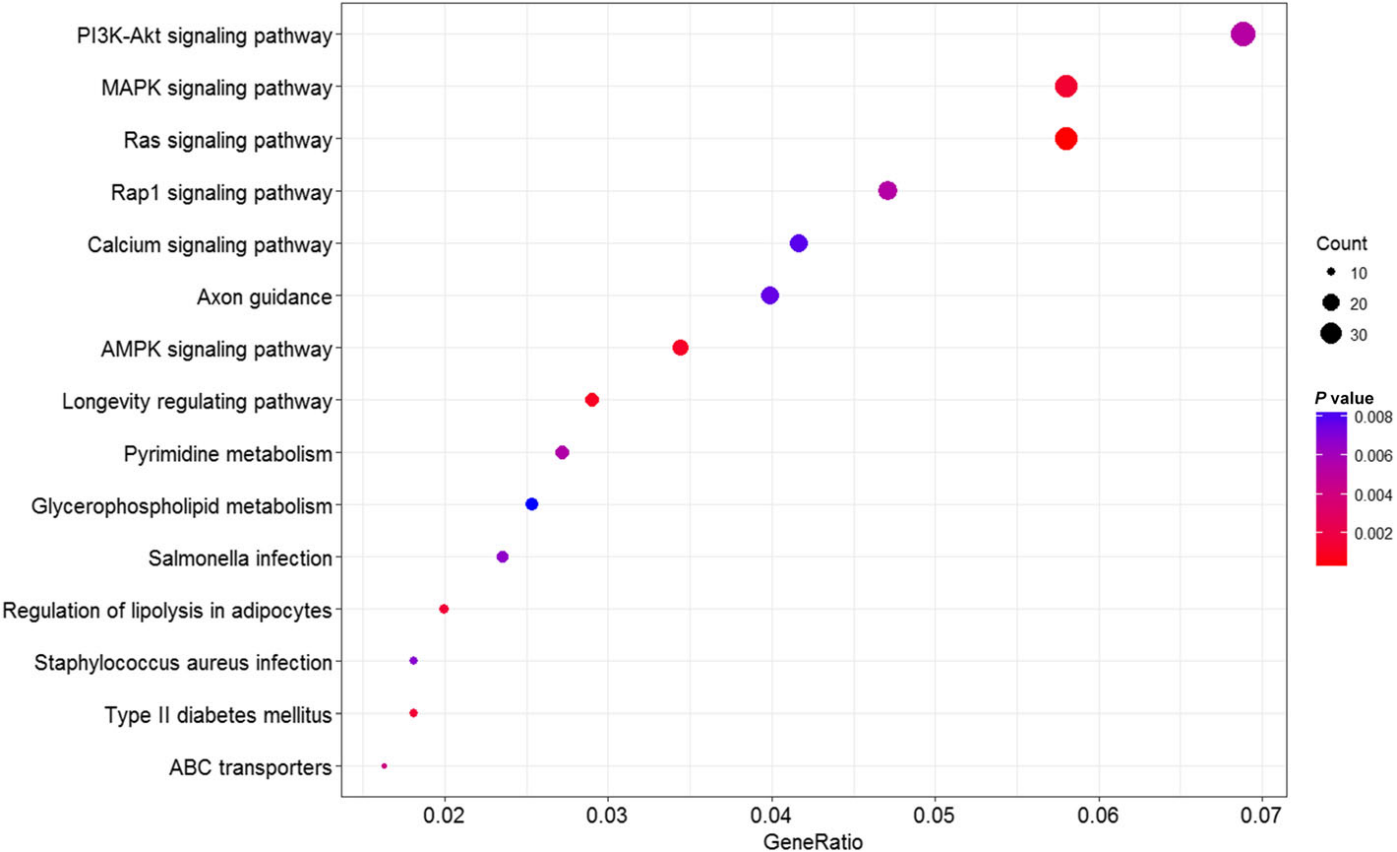
The KEGG functional annotation of target genes for TCONS_0007853. The sizes of the dots represent the counts of genes. The gene ratio indicates the ratio between the number of target genes associated with a KEGG term and the total number of genes in the KEGG term

In addition to the fact that location of a lincRNA within a QTL may suggest the function of the lincRNA in the QTL, we also used the LncTar to predict lincRNA-RNA interactions. To explore the functions of lincRNAs, 10 lincRNAs with the highest FPKM values were used to identify lincRNA-RNA interactions (Additional file 5). For each lincRNA, predicted target genes with the normalized free energy < −0.14 were identified and annotated by the GO and KEGG. GO functional annotation showed that five lincRNA target genes were enriched in different GO categories, such as biological processes of defense response, RNA phosphodiester bond hydrolysis, endonucleolytic and cAMP-mediated signaling, molecular function of oxidoreductase activity, cytokine receptor binding, protein transporter activity, cellular component of lysosome, membrane region, clathrin vesicle coat and intrinsic component of mitochondrial outer membrane (Additional file 6). For example, the target genes for TCONS_00162862 were involved in lipid transporter activity and ligase activity and fatty acid transport. On the other hand, target genes for TCONS_00162906, TCONS_00174720, TCONS_00078053, TCONS_00078723 and TCONS_00132942 were not enriched in any of the GO categories. As for the pathways, target genes for lincRNAs were enriched in several KEGG pathways such as the MAPK signaling pathway, calcium signaling pathway, glycosaminoglycan biosynthesis, PI3K-Akt signaling pathway, Insulin signaling pathway and Rap1 signaling pathway (Additional file 7). For example, target genes for TCONS_00010113 were enriched in the PI3K-Akt signaling pathway and Chemokine signaling pathway. Target genes for TCONS_0007853 were enriched in the Ras signaling pathway, Rap1 signaling pathway, MAPK signaling pathway and AMPK signaling pathway (Fig. 5, Additional file 7). However, target genes for TCONS_00026311, TCONS_00078723, TCONS_00132942, TCONS_00174720 and TCONS_00184509 were not annotated in any of the KEGG pathways.

### Selective validation of novel lincRNAs

**Fig. 6:**
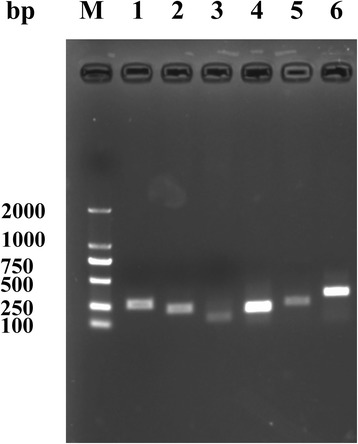
Validation of selected lincRNAs using PCR. Six randomly selected lincRNAs numbered from 1 to 6 are described in detail in Additional file 2

To confirm that our identified bovine lincRNAs were indeed expressed, six lincRNAs were randomly selected for PCR validation in vitro (Fig. 6). All PCR products had the expected size and our selected lincRNAs were amplified, suggesting that most, if not all, of our identified lincRNAs would be expressed in mammary gland tissues.

## Discussion

Long intergenic non-coding RNAs (lincRNAs) are a large and diverse class of transcribed RNA molecules with a length of more than 200 nucleotides that do not encode proteins. In the past few years, an increasing number of lincRNAs have been discovered in eukaryotic organisms, thanks to the advent of powerful and flexible RNA-seq methods. Despite the fact that only a few lincRNAs have been characterized experimentally in detail now, it is already known that they play regulatory and structural roles in almost every important biological process. Previous studies also used EST sequences to identify lncRNAs in cows [8]. However, such studies are incomplete, since ESTs sequences could not represent entire transcriptomes and reflect expression levels. On the other hand, RNA-seq can reveal dynamic transcriptome during different treatments or conditions and evaluate quantitative variations between different samples. Several studies have reported existence of bovine lincRNAs, mostly from non-mammary tissues.

In this study, we identified 184 bovine lincRNAs based on transcriptomic data from five RNA-seq datasets in bovine mammary gland tissues. The median expressed levels of lincRNA are low and only have about a tenth of the average expressed levels of mRNA [1]. Low expression levels of lincRNAs in transcripts present a challenge to distinguish them from thousands of one exon, low expression fragments and expression noise assembled from RNA-seq results [25]. In order to solve the problem, we have removed RNAs with only a single exon, the minimal read coverage threshold less than 3 or FPKM less than 1. While this process may remove some of lincRNAs with very low expression levels, thousands of unreliable fragments can be removed and only more reliable lincRNAs are kept. Similar strategies have been adopted in other studies. For example, Tang et al. annotated 300 human embryonic stem cell (hES) lincRNAs with FPKM >1 [26]. Similarly, Hangauer et al. selected FPKM > 1 as a criterion when they found tens of thousands lincRNAs in 127 human RNA-seq sequence files [27]. Using the stringent filter method, we identified 886 UITs as potential candidates for lincRNA.

To further identify true lincRNA, four programs including CPAT, CNCI, CPC and hmmscan were used collectively to calculate coding potentials of the 886 UITs. Accurate and quantitative assessment of coding potential is the most critical step in identifying lincRNAs. The CPC uses a support vector machine (SVM) learning classifier to identify six features extracted from input transcript sequences, while the CPAT is an alignment-free program and has used a logistic regression model to distinguish lincRNAs from noncoding sequences. A bovine special logistic regression model has been used [11]. CNCI is powerful software identifying coding or noncoding transcripts without known annotations. It extracts five features from the transcripts sequences and profiles adjoining nucleotide triplets (ANT) to discriminate coding transcripts from noncoding ones. The Hmmscan can search the lincRNA sequences against the hidden Markov model (HMM) database. We assume that the intersection of four software programs would predict lincRNA results more reliably and accurately than each single program. Consequently, 184 non-protein coding transcripts identified by all four programs were considered as lincRNAs. Approximately 42% of our 184 putative lincRNAs had a length ranging from 501 to 1500 bp, in agreement with those previously reported for other bovine tissues [10]. On the other hand, the number of our identified lincRNA was smaller than previous studies, possibly due to our stringent criteria. We feel that the stringent standards can filter out more false positive lincRNAs and lead to more reliable results.

Identification of lincRNAs in a specific tissue is the first step to understand biological functions of lincRNAs. Up to now, few studies have worked on the functions of lincRNAs in bovine. As a first step, we hypothesized that the location of lincRNAs on the chromosome could be suggestive for potential biological functions. Compared to the position of QTL in AnimalQTLdb, 36 different lincRNAs were found in 172 unique QTL regions associated with milk butyric acid percentage, milk capric acid percentage, milk iron content and milk protein content in milk quality and content. Another TCONS_00071212 is found in clinical mastitis QTL region (Additional file 4). These lincRNAs deserve particular attention in future studies. In addition, GO and KEGG analyses were performed for target genes of 10 lincRNAs with the highest FPKM values. We choose genes with the normalized free energy less than −0.14 for each of the 10 lincRNAs with the highest FPKM values. The number of genes was chosen to illustrate the concept that GO and KEGG analyses can predict presumptive functions for lincRNAs.

Target genes of TCONS_00010113 were significantly enriched in cellular component of clathrin coat and clathrin vesicle coat. Clathrin-coated vesicle were presented in the rabbit lactating mammary gland and involved in the biogenesis of casein-containing secretory vesicles and transcytotic pathway [28]. Intriguingly, KEGG pathway annotation showed that target genes for TCONS_00010113 were enriched in the PI3K-Akt signaling pathway. By blocking the PI3K-Akt signaling pathway using TK1258, Dey et al. induced apoptosis in breast cancer model cell lines 4T1 [29]. Target genes for TCONS_0007853 were enriched in Rap1 signaling pathway and MAPK signaling pathway. Itoh et al. found that the Rap1 was a pivotal element in organizing acinar structure and inducing lumen formation in HMT-3522 human mammary epithelial cells. Increased activation of Rap1 induced tumor formation and progression to malignancy [30]. Four classes of MAPK signaling pathway, including the c-Jun N-terminal kinase (JNK) pathway, the ERK5 pathway, extracellular regulated kinase (ERK)1/2 pathway and the p38 pathway, functioned in mammary epithelial cells and involved in breast disease [31]. Based on these published results and the KEGG pathway, it is clear that target genes for TCONS_0007853 could be involved in development of bovine mammary gland in bovine mammary gland and their roles need to be confirmed.

Analyzing the molecular basis for QTLs is a major challenge. Precisely identifying and verifying causative genes for production traits are still very complicated and difficult, considering that most traits are polygenic. Grisart et al. mapped a QTL region and identified DGAT1 on chromosome 14 as a major gene for milk fat content [32]. However, such a causative relationship is still not available for almost all other traits. Our results reveal that some of the 184 lincRNAs are located within QTL regions. Assuming that some lincRNAs can act on their neighboring gene expression and we can predict functions for lincRNAs accurately, we may be able to use lincRNAs to locate candidate genes for production traits. The *cis* relationship between lincRNAs and their target genes have been reported previously. For example, Faghihi et al. identified an lincRNA called b-secretase-1 antisense transcript (*BACE1*-AS), which regulates BACE1 mRNA and protein expression in vitro. BACE1 is an important enzyme in Alzheimer’s disease [33]. Their results indicate the involvement of *BACE1*-AS in Alzheimer’s disease. Similarly, Modarresi et al. found brain-derived neurotrophic factor (BDNF) BDNF, a factor involved in the Huntington’s disease, was repressed by an lncRNA BNDF antisense RNA transcript (*BDNF*-AS) [34].

Our current results can provide interesting insight into the interaction between lincRNAs and QTL related marker-assisted selection genes in cows. Of course, finding the relationship between lincRNAs and QTL genes need other techniques. RNA fluorescent in situ hybridization (FISH) techniques can enable the absolute measurement of lincRNA transcript abundance and detect precise subcellular localization [35, 36]. Understanding the cellular and molecular mechanism of lincRNAs also relies on identification of RNA-protein and RNA-DNA interactions. RNA immunoprecipitation (RIP) and ultraviolet cross-linking and immunoprecipitation (CLIP) method are able to identify RNA-protein interactions [37, 38]. Chromatin isolation by RNA purification (ChIRP) and capture hybridization analysis of RNA targets (CHART) can be used to map the DNA binding sites for lincRNAs [39, 40]. These methods as well as more research on lincRNAs will certainly lead to better understanding the mechanism of lincRNAs and guide our future experimental designs for finding causative genes in QTLs for milk yield and milk quality as well as for mastitis resistance.

## Conclusion

In this study, 184 lincRNAs including 112 novel ones were identified in bovine mammary glands. In addition, 113 different lincRNAs were located within 399 unique different cattle QTL regions. In particular, several lincRNAs were placed in QTLs affecting clinical mastitis, milk quality or production and their involvement in these parameters is worthy of further investigation. In addition, the identification of novel lincRNAs significantly expanded the repertoire of lincRNAs for dairy cattle and consequently will help understand functions of the bovine lincRNAs.

## Additional files

Additional file 1: RNA-Seq datasets used in this study. (XLSX 10 kb)

Additional file 2: Primer sequences used for RT-PCR. (DOCX 14 kb)

Additional file 3: List of lincRNA information identified in bovine mammary glands of this study; Additional file 3a. List of 112 novel lincRNA sequences; Additional file 3b. List of 72 known lincRNA sequences, Additional file 3c. GTF file of 184 lincRNAs. (XLSX 114 kb)

Additional file 4: List of identified lincRNAs located within known QTL regions. (XLSX 38 kb)

Additional file 5: Predicted target genes for 10 lincRNAs with the highest FPKM values; Additional file 5a. Predicted target genes for 10 lincRNAs with the highest FPKM values by LncTar software; Additional file 5b: The neighbouring coding genes (30 K upstream and downstream) of 10 lincRNAs. (XLSX 143 kb)

Additional file 6: Gene ontology (GO) functional annotations for target genes of 10 lincRNAs with the highest FPKM values. The *P* value cut-off <0.05 and *q* value cut-off <0.2 were adopted to include the target genes. (XLSX 23 kb)

Additional file 7: KEGG pathway annotation results for target genes of 10 lincRNAs with the highest FPKM values. The *P* value cut-off <0.05 and *q* value cut-off <0.2 were adopted to include the target genes. (XLSX 14 kb)

## Abbreviations

FPKM: Fragments per kilobase of transcript per million fragments mapped
GO: Gene ontology
KEGG: Kyoto encyclopedia of genes and genomes
lincRNAs: Intergenic lncRNAs
QTL: Quantitative Trait Loci
UITs: Unknown intergenic transcripts
