# Supplementary material for: Identification and characterization of long intergenic noncoding RNAs in bovine mammary glands
Source: BMC Genomics. 2017 Jun 19;18:468. doi: 10.1186/s12864-017-3858-4 (PMC5477121; doi:10.1186/s12864-017-3858-4)
Supplement: Supplementary file 2 — Primer sequences used for RT-PCR. (DOCX 14 kb) [file 12864_2017_3858_MOESM2_ESM.docx]

|  | **Transcript_ID** | **Forward primer sequence (5'-3')** | **Reverse primer sequence(5'-3')** | **Amplicon size (bp)** | **annealing temperature(℃)** |
| --- | --- | --- | --- | --- | --- |
| 1 | TCONS_00151364 | GCCTCCTCCATCCGTTCA | CCGCCATCCAGAGTCTACAAA | 277 | 64 |
| 2 | TCONS_00082878 | AAACAAGAGCAAGAATCCCAGAG | CCAAGGCCCAGAACTTAGCAG | 233 | 64 |
| 3 | TCONS_00046420 | CTCTTGAACTACACCACCCT | CTCTTGAACTACACCACCCT | 206 | 62 |
| 4 | TCONS_00187831 | AGGATTGGGAGGCTCTG | CTTGCTACTGGGTGTCTATG | 211 | 60 |
| 5 | TCONS_00143228 | CTTGTGAGATGCAGGAA | GTAGTTATGGGTGGGTTT | 257 | 60 |
| 6 | TCONS_00080413 | GAGGAGGAGCAGTGGGT | AAGACAGGGAAGCAGGTA | 336 | 60 |

**Additional file 2： Primer sequences used for RT-PCR**
